# Short-Term: Cellular Metabolism and Gene Expression During the Onset of Diabetic Kidney Disease: A Diabetes Mellitus Experimental Model

**DOI:** 10.3390/ijms26199676

**Published:** 2025-10-04

**Authors:** Jéssica Encinas, Glaucia Veiga, Joyce Raimundo, Matheus Perez, Giuliana Petri, Renan Cavalheiro, Pedro Reis, Laura Maifrino, Beatriz Alves, Fernando Fonseca

**Affiliations:** 1 Laboratório de Análises Clínicas, Centro Universitário FMABC, Santo André 09060-870, Brazil; araujo.jfreitas@gmail.com (J.E.); joyce.regina@gmail.com (J.R.); matheusmpontoperez@gmail.com (M.P.); pedro.reis@aluno.fmabc.net (P.R.); bcaalves@uol.com.br (B.A.); profferfonseca@gmail.com (F.F.); 2 Coordenação da Comissão de Ética no uso de Animais, Centro Universitário FMABC, Santo André 09060-870, Brazil; giuliana.petri@fmabc.br; 3 Departamento de Morfologia e Fisiologia, Centro Universitário FMABC, Santo André 09060-870, Brazil; renan.cavalheiro@fmabc.net; 4 Laboratório de Estudos Morfoquantitativo e Imunohistoquímica (LEMI), Departamento Morfologia, Universidade São Judas Tadeu, São Paulo 03166-000, Brazil; lmaifrino@uol.com.br; 5 Departamento de Ciências Farmacêuticas, Universidade Federal de São Paulo, Campus Diadema, Diadema 09972-270, Brazil

**Keywords:** biomarkers, diabetes complications, collagen deposition

## Abstract

Diabetes is a chronic disease with a rising global prevalence. Research focuses on understanding its metabolic implications and early signaling of disease onset and complications, particularly the interconnected effects on the kidneys and brain. The objective of this study was to evaluate the expression profile in the genes *Mct1*, *Mct4*, *Cd147*, *Hif-1α* and *Vegf* for different biological matrices in rats induced to diabetes in the determined periods of 7, 21, 30 and 40 days. Methods: Wistar rats (160–180g, *n* = 68), divided into sham and diabetic groups, were evaluated according to tissue samples from the brain and kidney, using classical biochemical analyses and assessing temporal intergroup differential gene expression by qPCR. Additionally, immunohistochemical analysis was performed on kidney samples to evaluate collagen deposition. In the renal tissues, we observed a decrease in the expression of *Hif-1α* (21 vs. 30 days) and *Vegf* (21 vs. 40 days), accompanied by an increase in collagen deposition. In the brain, alterations were observed in all evaluated genes when comparing the early group (7 days) to the later groups (30 and 40 days). We observed that the evaluated genes, as well as the collagen deposition analyzed by immunohistochemistry, are related to metabolic changes that, over time, contribute to the worsening of diabetes and the progression of secondary diseases directly and/or indirectly involving the studied tissues.

## 1. Introduction

Diabetes is a chronic metabolic disease characterized by hyperglycemia; of multiple origins, it results from a deficiency in the production and/or action and/or secretion of insulin. Epidemic in nature, this disease has shown an alarming rise and prevalence in recent decades, affecting approximately 589 million adults worldwide in 2025 [1,2]. Moreover, late diagnosis of diabetes and the development of secondary pathologies contribute to an increase in the morbidity and mortality of these patients [2]. Studies reveal that the metabolic disturbance caused by diabetes can mainly trigger diabetic kidney disease (DKD), diabetic neuropathy, and other complications [2,3,4].

At the cellular level, diabetes profoundly alters glucose metabolism and energy homeostasis. Under normal physiological conditions, insulin promotes glucose uptake primarily in muscle and adipose tissues, facilitating glycolysis, glycogenesis, and lipogenesis. However, in the context of insulin deficiency or resistance, as seen in diabetes, cells shift their energy metabolism towards alternative substrates, such as fatty acids and amino acids, resulting in enhanced β-oxidation, proteolysis, and gluconeogenesis [5,6]. This metabolic shift leads to the increased production of reactive oxygen species (ROS), mitochondrial dysfunction, and the activation of inflammatory pathways, contributing to cellular stress and apoptosis [7,8,9].

Hyperglycemia also exacerbates oxidative stress and alters key metabolic sensors, such as AMPK (AMP-activated protein kinase) and mTOR (mechanistic target of rapamycin), disrupting cellular adaptation to energy supply and demand [10,11].Additionally, chronic exposure to high glucose impairs insulin signaling via the PI3K/Akt pathway and increases the activity of the hexosamine and polyol pathways, leading to glycation of proteins and lipids and activation of pro-fibrotic and pro-inflammatory cascades [12,13]. These biochemical and molecular events play a pivotal role in the pathogenesis of diabetic complications, particularly in metabolically active tissues such as the kidney and brain.

Among the advances of the last decades, referring to the diagnosis and prognosis of this disease, there was an understanding of the pathophysiological processes and the different metabolic variations in the evolution of diabetes through molecular markers [14]; however, there is still a need for a panel of markers that allows us to monitor changes related to the different organs affected as the disease progresses. Biomarkers are biochemical, molecular, or histological indicators used for the assessment of metabolic and molecular dysfunctions in pathological and/or pharmacological processes [14]. In this study, we aim to evaluate five distinct biomarkers involved in tissue oxidation, angiogenesis, and glucose metabolism biological processes.

Among these, monocarboxylate transporters (MCTs) play a crucial role. These membrane proteins are primarily responsible for transporting lactate, pyruvate, and ketone bodies across the plasma membrane [5]. The MCT family includes 14 isoforms with diverse, yet not fully elucidated, transport characteristics and distinct tissue distribution [6]. *MCT1* is the most widely expressed isoform, being active in multiple tissues [6,7], whereas *MCT4* is predominantly found in cells with high glycolytic rates [8]. Notably, *MCT4*’s expression and function are dependent on the co-expression of cluster of differentiation 147 (*CD147*), also known as extracellular matrix metalloproteinase inducer (EMMPRIN), a membrane glycoprotein responsive to metabolic stimuli [9]. *CD147* expression is regulated by cellular metabolic activity, supporting glycolysis and cellular respiration [8].

MCT activity is also modulated by oxygen availability via hypoxia-inducible factors (HIFs), particularly *HIF-1α*, which is broadly expressed in mammalian tissues [10,11]. In hypoxic conditions, HIF-1α becomes activated, initiating adaptive metabolic responses that are still not fully understood in the context of diabetes [12,13]. These conditions also influence the expression of vascular endothelial growth factor (*VEGF*), a dimeric glycoprotein with potent angiogenic activity that supports endothelial cell migration and inhibits apoptosis [15,16].

The development of diabetic complications involves early molecular changes, including metabolic, oxidative, and fibrotic alterations. Therefore, this study primarily sought to evaluate the temporal expression of key genes related to specific pathways, such as hypoxia (*Hif-1α*, *Vegf*), metabolic transport (*Mct1*, *Mct4*, *Cd147*), and cellular stress, specifically in the brain and kidney during the early stages of diabetes.

In this study, we investigate the interaction between the bulbar region of the brainstem and renal function through specific molecular markers and their interrelations. One of the aims of this study is to evaluate the kidney–brain axis in the maintenance and progression of renal diseases resulting from chronic hyperglycemia. This focus is justified by evidence in the literature indicating that the bulbar region, particularly the vasomotor center located in the rostral ventrolateral medulla (RVLM) that modulates sympathetic activity that directly influences the kidneys [17,18]. This modulation occurs via regulation of sympathetic preganglionic neurons in the spinal cord, which project to the celiac plexus and innervate the kidneys [19,20]. Through this pathway, sympathetic activity controls renal vasculature, renin release by the juxtaglomerular apparatus, and tubular reabsorption, thereby adjusting renal function according to the organism’s metabolic and cardiovascular demands [21].

Due to the scarcity of studies on effective markers capable of identifying the onset of metabolic alterations in diabetes, this study primarily aimed to evaluate the expression profile of the *Mct4*, *Cd147*, *Hif-1α* and *Vegf* genes in diabetic rats, in target tissues such as brain and kidney, in different periods after the onset of the disease (7, 21, 30 and 40 days). The selected timepoints were chosen to monitor the dynamic progression of these changes, prior to the manifestation of overt histopathological damage. As a secondary objective, the selected time points were chosen to monitor the dynamic progression of these changes before the onset of overt histopathological damage.

## 2. Results

Sixty-eight Wistar rats were utilized and assigned to their respective experimental groups (DM or NDS). The diabetogenic agent alloxan, administered intraperitoneally (120 mg/kg), effectively induced diabetes in the DM group. This compound exhibits selective cytotoxicity toward pancreatic β cells, promoting pancreatic islet degeneration and the onset of diabetes, while demonstrating high specificity of action and a low mortality rate [22,23]. In our study, the DM group had a mortality rate ~21%. Animals in the DM group exhibited a reduction in the delta parameter, reflecting significant weight differences (g) compared to their respective control groups. Initial body weight was recorded on the day of induction (alloxan or sham) and reassessed following DM confirmation. In parallel with these systemic changes, a significant increase in the relative weight (tissue weight/body weight) of the right kidney was observed in the diabetic groups compared to their respective controls on the day of euthanasia. This difference was statistically significant in most of the evaluated groups, with the following values: group 7 (DM7 0.43 ± 0.06, *n* = 13 vs. NDS7 0.33 ± 0.07, *n* = 11, * *p* = 0.02), group 21 (DM21 0.61 ± 0.09, *n* = 7 vs. NDS21 0.35 ± 0.03, *n* = 10, * *p* = 0.0001), group 30 (DM30 0.55 ± 0.25, *n* = 8 vs. NDS30 0.35 ± 0.07, *n* = 6, *p* = 0.06), and group 40 (DM40 0.69 ± 0.14, *n* = 5 vs. NDS40 0.39 ± 0.05, *n* = 7, * *p* = 0.0004).

### 2.1. Biochemical Parameters

The animals in the DM group exhibited blood glucose levels exceeding 250 mg/dL, which were statistically higher than those in the NDS group. According to established normality parameters [24,25], these findings confirm the experimental model used. Regarding the data assessing renal function, all groups exhibited values within the reference range, except for urea.

Plasma markers reflecting potential renal impairment during the onset of DM were analyzed (Table 1). All factors analyzed showed statistically higher biochemical concentrations in the DM group compared to the NDS group. A significant increase in plasma urea levels, exceeding the reference values for rats, was observed in the following groups: group 7 (DM7 97.94 ± 28.27, *n* = 11 vs. NDS7 57.43 ± 14.71 mg/dL, *n* = 8 * *p* = <0.0002), group 21 (DM21 153.38 ± 44.13, *n* = 5 vs. NDS21 68.31 ± 5.08, *n* = 10 mg/dL, * *p* = <0.0001), group 30 (DM30 170.20 ± 21.00, *n* = 6 vs. NDS30 79.62 ± 7.15, *n* = 5 mg/dL, * *p* = <0.0007), and group 40 (DM40 173.10 ± 42.71, *n* = 5 vs. NDS40 82.52 ± 12.00, *n* = 5 mg/dL, * *p* = <0.001). Despite the observed alteration, these findings suggest that diabetic nephropathy did not manifest in the present model.

### 2.2. Gene Expression Profiling of the Analyzed Targets

**Figure 1:**
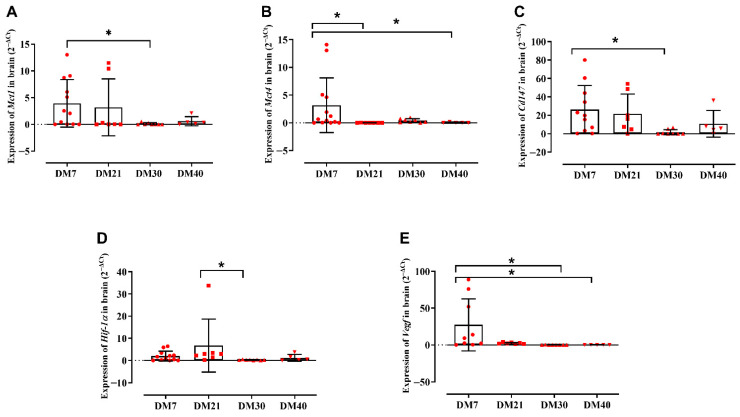
Relative gene expression of markers involved in hypoxia, inflammation, and vascular remodeling in the bulbar region of diabetic rats. Quantitative PCR (qPCR) analysis was performed to assess the expression of (**A**) *Mct1*, (**B**) *Mct4*, (**C**) *Cd147*, (**D**) *Hif-1α*, and (**E**) *Vegf* in brain tissue at 7, 21, 30, and 40 days after induction of diabetes (DM7, DM21, DM30, and DM40). Results are expressed as 2^–ΔCt values. Data are shown as mean ± standard deviation (SD); red symbols represent individual animals. Statistically significant differences were identified using Dunn’s multiple comparisons test following Kruskal–Wallis analysis (* *p* < 0.05).

During the onset of DM, significant molecular alterations were observed in the brain, particularly in the expression of key biomarkers. Gene expression analysis revealed marked differences in *Mct1, Mct4, Cd147, Hif-1α*, and *Vegf* across different time points. Data were analyzed using Dunn’s multiple comparisons test to assess differences among diabetic groups at different time points. A statistically significant reduction of *Mct1* expression was observed between the DM7 and DM30 groups (mean rank difference = 10.42; * *p* = 0.0447), indicating a decrease in the measured variable at day 30 compared to day 7. No significant differences were found between DM7 and DM21 (mean rank difference = 4.988; *p* = 0.7893) or between DM7 and DM40 (mean rank difference = 4.617; *p* > 0.9999). These findings are consistent with the mean values observed across groups, with a marked decline from 3.936 in DM7 to 0.1163 in DM30. *Mct4* expression was significantly decreased at both the early (DM7: 7.85 ± 9.80, *n* = 13 vs. DM21: 3.18 ± 5.33, *n* = 8; * *p* < 0.05) and late stages (DM7: 7.85 ± 9.80, *n* = 13 vs. DM40: 0.30 ± 0.43, *n* = 5; * *p* < 0.05) of the disease. In addition, *Cd147* expression was markedly reduced in the late stage (DM7: 18.90 ± 20.20, *n* = 11 vs. DM30: 1.62 ± 2.77, *n* = 8; * *p* < 0.05) (Figure 1).

**Figure 2:**
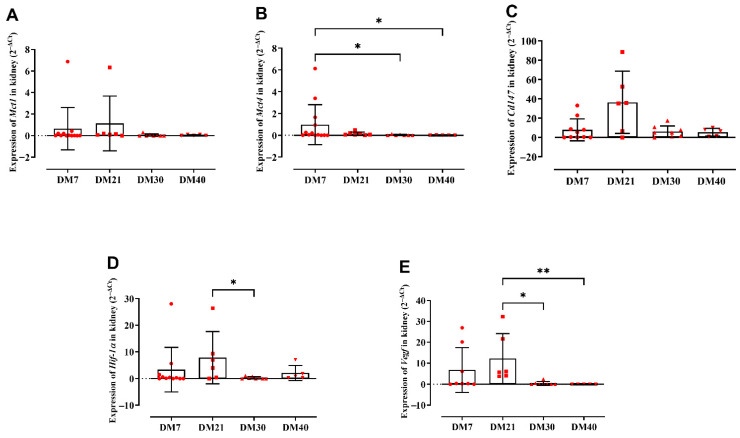
Relative gene expression of markers involved in hypoxia, inflammation, and vascular remodeling in the kidney. Quantitative PCR (qPCR) analysis was performed to assess the expression of (**A**) *Mct1*, **(B**) *Mct4*, (**C**) *Cd147*, (**D**) *Hif-1α*, and (**E**) *Vegf* in kidney tissue at 21, 30, and 40 days after induction of diabetes (DM21, DM30, and DM40). Data are shown as mean ± standard deviation (SD). Statistically significant differences were identified using Dunn’s multiple comparisons test following Kruskal–Wallis analysis (* *p* < 0.05, ** *p* < 0.01).

In parallel, a marked decrease was also observed in the expression of *Hif-1α* (DM21: 6.73 ± 11.97, *n* = 7 vs. DM30: 0.25 ± 0.19, *n* = 8; * *p* ≤ 0.05) (Figure 2A). *Vegf* expression showed a decline in the 7-day group (DM7: 17.36 ± 27.53, *n* = 9) compared to both the 30-day (DM30: 0.08 ± 0.12, *n* = 8; * *p* = < 0.05) and 40-day groups (DM40: 0.11 ± 0.08, *n* = 5; * *p* = < 0.05) as well as between the 21-day and 30-day groups (DM21: 2307 ± 1064, *n* = 8 vs. DM30: 0.08 ± 0.12, *n* = 8, * *p* = < 0.05). A significant decrease in *Hif-1α* expression was evident between the 21-day and 30-day groups (DM21: 7.85 ± 9.80, *n* = 6 vs. DM30: 0.30 ± 0.43, *n* = 8, * *p* = < 0.05). Similarly, *Vegf* expression showed a marked decline from the 21-day group (DM21: 28.19 ± 43.48, *n* = 7) to both the 30-day (DM30: 0.44 ± 0.86, *n* = 8) and 40-day groups (DM40: 0.01 ± 0.01, *n* = 5, * *p* ≤ 0.05) (Figure 1).

Gene expression analysis in renal tissue of diabetic rats revealed significant temporal changes in markers related to hypoxia and tissue remodeling. The expressions of *Mct1* (A) and *Mct4* (B) showed no significant difference between the groups (*p* > 0.05). Renal expression of the *Mct4* gene in diabetic rats revealed statistically significant differences between the early and later stages of the disease. The DM7 group exhibited a mean expression of 3.936 ± 4.440 *n* = 13, which declined progressively over time. By DM21, the mean expression decreased to 3.185 ± 5.327, *n* = 9 though this reduction was not statistically significant compared to DM7 (*p* > 0.9999). In contrast, a marked reduction was observed in the DM30 (0.1163 ± 0.1998, *n* = 8 *p* = 0.0412) and DM40 (0.6028 ± 0.8601, *n* = 5 *p* = 0.0374) groups, these reductions were statistically significant when compared to DM7. These findings indicate a downregulation of *Mct4* expression in renal tissue as diabetes progresses, possibly reflecting diminished metabolic demand or alterations in lactate transport activity in the kidney. The analysis of *Cd147* gene expression in renal tissue of diabetic rats showed no statistically significant differences among the experimental groups at different time points.

The renal expression of *Hif-1α* varied across the experimental time points following diabetes induction. At 7 days (DM7), the mean expression level was 3.331 ± 8.358, *n* = 11. At 21 days (DM21), a peak in expression was observed (7.846 ± 9.792, *n* = 6), although this increase was not statistically significant when compared to DM7 (*p* = 0.7893). By 30 days (DM30), a marked reduction in gene expression was evident (0.3039 ± 0.4253, *n* = 8), and this decrease was statistically significant compared to DM7 (*p* = 0.0447). At 40 days (DM40), the expression level rose slightly to 2.072 ± 2.842, *n* = 5, but this change was not significant in relation to DM7 (*p* > 0.9999). Regarding *Vegf* (E), its expression levels were significantly higher in the DM7 (6.787 ± 10.690, *n* = 9) and DM21 (12.271 ± 11.923, *n* = 7) groups compared to DM30 (0.436 ± 0.866, *n* = 8, *p* < 0.05) and DM40 (0.010 ± 0.014, *n* = 5, *p* < 0.01), indicating a later suppression of this gene’s expression (Figure 2).

### 2.3. Picrosirius Analysis of Collagen Deposition in Renal Tissue

**Figure 3:**
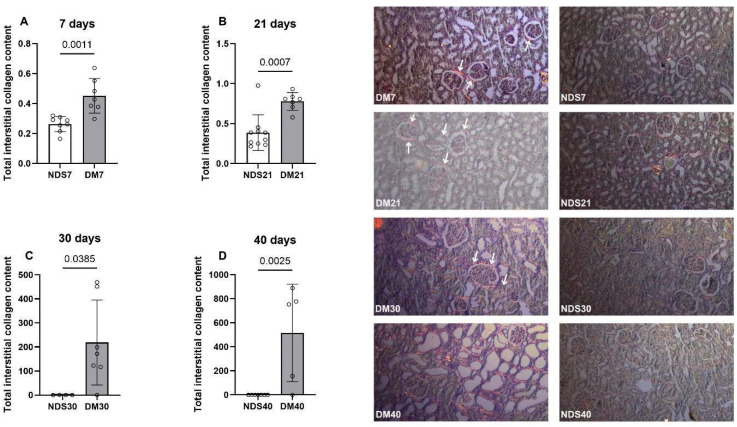
Quantification and histological analysis of interstitial collagen deposition (types I and III) in renal tissue at different time points following diabetes induction. (**A**–**D**) Bar graphs showing the total interstitial collagen content in control (NDS) and diabetic (DM) groups at 7, 21, 30, and 40 days post-induction. Right panels: Representative photomicrographs of kidney sections stained with Picrosirius Red, highlighting interstitial collagen fibers (red/orange birefringence under polarized light). White arrows indicate areas of marked collagen accumulation, predominantly in the interstitial compartment of diabetic animals (DM7–DM40), with progressive intensity over time. Scale bars = [100 µm].

Quantification of total interstitial collagen content revealed a significant increase in diabetic groups compared to non-diabetic controls at all evaluated time points. At 7 days, the DM7 group showed a higher mean collagen content (0.4514 ± 0.1156, *n* = 7) than the NDS7 group (0.2631 ± 0.0517; *n* = 8, *p* = 0.0011). This trend persisted at 21 days, with DM21 animals presenting increased collagen levels (0.7770 ± 0.1110, *n* = 7) compared to NDS21 (0.3870 ± 0.2227; *n* = 9, *p* = 0.0007). At 30 days, collagen accumulation was further elevated in the DM30 group (218.9 ± 176.4, *n* = 7) versus NDS30 (0.3330 ± 0.1214; *n* = 4 *p* = 0.0385). Similarly, at 40 days, the DM40 group exhibited a marked rise in interstitial collagen content (515.1 ± 405.5, *n* = 5) relative to NDS40 (0.2931 ± 0.1092; *n* = 7 *p* = 0.0025). Histological analysis using Picrosirius Red staining confirmed these findings, showing more intense collagen fiber deposition in the interstitial space of diabetic kidneys, especially at later stages (Figure 3).

## 3. Discussion

This study analyzed the expression of *Mct1, Mct4, Cd147, Hif-1α*, and *Vegf* in the brainstem and kidneys of diabetic rats. Alloxan effectively induced diabetes without overt nephropathy, though elevations in urea and creatinine indicated early renal dysfunction. In the brainstem, the consistent downregulation of *Mct1* and *Mct4* suggests impaired monocarboxylate transport and astrocytic dysfunction, compromising neuronal energy supply and sympathetic regulation. *Mct1* downregulation likely disrupts lactate uptake in neurons, a known alternative substrate during energetic stress, while decreased *Mct4* may reflect astrocyte dysfunction, previously linked to central autonomic failure in diabetes [24,25,26,27,28,29,30,31,32,33].

Molecular analysis showed reduced expression of *Mct1, Mct4, Hif-1α, Cd147*, and *Vegf* in the bulbar region of diabetic rats. MCTs regulate the cellular transport of key metabolites such as pyruvate, lactate, butyrate, and ketone bodies, thus influencing energy metabolism. Insulin deficiency disrupts brain energy homeostasis and function [28,29]. In diabetes, hyperglycemia impairs glucose uptake and energy production, leading to altered monocarboxylate metabolism and compromised neuronal function.

The reduction in *Mct1* expression observed in the bulbar region of diabetic rats may reflect an adaptive or pathological alteration in cerebral energy metabolism in response to hyperglycemia. Silva et al. (2000) [30] demonstrated that *Mct1* expression is decreased in adipocytes of streptozotocin-induced diabetic rats, suggesting that hyperglycemia may directly affect the gene and functional regulation of these transporters. In the brain, Pierre et al. (2007) [31] highlight that MCT1 plays a key role in maintaining energy homeostasis, and its alteration may negatively impact the uptake of glucose-alternative substrates such as lactate, particularly in regions involved in autonomic integration, such as the brainstem. In this context, the reduction in *Mct1* activity may contribute to local metabolic imbalance and, potentially, to functional alterations in bulbar nuclei.

Mct4 in the bulbar region is essential for energy homeostasis and autonomic regulation of the central nervous system (CNS). The observed reduction in its expression may indicate astrocytic dysfunction, impairing neural signaling and contributing to brain functional decline [31,32,33]. This decrease correlates with diabetes progression, suggesting impacts on energy and neuromodulatory homeostasis. The bulbar region contains premotor neurons of the sympathetic nervous system, which regulate vascular tone and cardiovascular function; thus, alterations in MCTs, specially *Mct4*, may cause autonomic dysfunction induced by hyperglycemia. Studies show that reduced *Mct2* expression in the hypothalamus and hippocampus is linked to hyperglycemia and brain dysfunction [34,35]. Halestrap et al. (2013) [7] emphasize that glucose metabolism is directly related to MCT function, particularly isoform 4.

The CD147 plays a crucial role in cellular energy metabolism by regulating the expression and activity of MCTs, facilitating substrate transport across cell membranes [8,9]. The diabetic environment induces diverse and well-documented alterations, including increased reactive oxygen species (ROS) production [36,37], activation of pro-inflammatory cytokines [38], and inflammatory pathways [37]. These changes are directly related to insulin deficiency and disrupt specific metabolic pathways, causing alterations in the expression of interconnected genes. This process negatively regulates several genes, including *Cd147* and *Vegf*, which are critical for energy homeostasis and cellular adaptation. Under hyperglycemic conditions, *Cd147* expression can be modified, leading to activation or inhibition of matrix metalloproteinases (MMPs) and resulting in extracellular matrix dysfunction and vascular damage [39]. The observed decrease in *Cd147* expression at 30 days coincides with reduced *Mct4* expression and diminished *Hif-1α* activity, as supported by our data and previous studies [40]. This negative regulation is linked to increased prolyl hydroxylase domain (PHD) activity, which suppresses Hif-1α expression [41]. The authors suggest that suppression of gene activity is linked to the adaptive and energetic failure of brain cells, triggering severe pathological processes and contributing to diabetic neuropathy.

Hif-1α plays a central role in regulating *Vegf* and *Cd147* expression, a relationship confirmed in our study through correlated gene modulation across disease progression. *Vegf* expression, primarily controlled by Hif-1α, is inhibited by increased ROS, which disrupt angiogenic signaling [42]. Mi et al. (2019) [43] showed in a diabetes model that reduced *Hif-1α/Vegf* expression is associated with neurodegeneration and endothelial dysfunction, accelerating vascular disorders and contributing to brain hemorrhages and stroke. In our study, the most significant gene expression decreases occurred in the 30-day group, suggesting stage-specific tissue modulation linked to neurovascular alterations in diabetes.

Our data suggest a novel feedback mechanism in diabetic conditions where the early downregulation of *Mct4* precedes and may contribute to the subsequent decrease in Hif-1α expression. While *Hif-1α* is widely recognized as a key transcriptional regulator of *Mct4* under metabolic stress [7], this disrupted regulatory axis in the diabetic brain highlights potential metabolic maladaptation’s. Such dysregulation may impair cellular energy homeostasis and exacerbate neurovascular dysfunction during diabetes progression. Further research is needed to elucidate the precise molecular pathways underlying this feedback and its implications for diabetic neuropathy.

Our current data highlight substantial alterations in the brainstem region, suggesting early metabolic damage that may lead to profound functional and structural changes. These findings emphasize metabolic abnormalities that serve as precursors in the onset and progression of diabetic neuropathy over time. In line with this, Tesfaye et al. (1996) [44] previously described the association between elevated blood glucose levels and the induction of hypoxia in nerve fibers. This interaction contributes to impulse generation, leading to subsequent functional and structural transformations associated with diabetic neuropathy.

This study investigated gene expression interactions in specific brain regions, focusing on the bulbar region, which is metabolically crucial for sympathetic regulation [45,46]. We also analyzed renal gene expression to assess alterations within the renin-of RAAS [47]. Our results revealed significant changes in *Hif-1α* and *Vegf* expression in both kidney and brain, predominantly in late-stage experimental groups, suggesting a shared regulatory mechanism between these organs under diabetic conditions. Modulation of RAAS-related genes, including angiotensin II and aldosterone, may simultaneously influence renal pathology and central nervous system function, highlighting the systemic nature of diabetes-induced dysfunction [47].

DM involves early pathophysiological changes, including oxidative stress, inflammation, apoptosis, and mitochondrial dysfunction, which drive organ-specific complications. Metabolic dysregulation affects both the central nervous system and the kidney, contributing to microvascular dysfunction and fibrosis progression. In this study, dysregulated genes can be linked to canonical pathways such as ROS production, *NF-κB* and *NLRP3* inflammasome signaling, apoptotic mechanisms, and impaired AMPK signaling, highlighting their role in tissue injury, insulin resistance, and broader metabolic dysregulation [48,49,50].

The current results highlight the interconnection between the regulation of *Hif-1α*, *Vegf*, and *Mct*, emphasizing that imbalance among oxidative stress, inflammation, and hypoxia contributes to fibrosis and microvascular dysfunction in metabolically vulnerable organs like the kidney and central nervous system. Sustained high glucose levels and chronic inflammation activate nuclear factor kappa B and protein kinase C signaling pathways, promoting mitochondrial dysfunction and reducing *Hif-1α* expression, thereby worsening endothelial dysfunction and renal damage [51,52]. This cascade ultimately results in progressive endothelial impairment and microangiopathy, reducing renal blood flow and exacerbating local ischemia.

The observed reduction in *Hif-1α* gene expression directly leads to the downregulation of *Vegf*, a key factor in vascular angiogenesis essential for maintaining renal function under stress [53,54,55]. This decline compromises glomerular perfusion and vascular protection, leaving renal metabolism vulnerable. The decreased *Vegf* expression impairs endothelial and podocyte cell integrity, increasing glomerular permeability and initiating renal alterations that may trigger diabetic nephropathy [56]. Furthermore, the simultaneous reduction of *Hif-1α* and *Vegf* diminishes the kidney’s angiogenic capacity, exacerbating tissue hypoxia and promoting fibrosis [57]. This vicious cycle accelerates renal function loss, contributing to the progression of diabetic kidney disease and its complications.

Bohuslavova et al. (2017) [58] and Wang et al. (2014) [59] reported decreased *Hif-1α* expression in diabetic animals, associating this reduction with structural damage in the renal cortex and podocytes, highlighting impaired kidney adaptation to hypoxia under chronic hyperglycemia and oxidative stress. Chen et al. (2016) [60] further demonstrated that silencing *Hif-1α* leads to reduced *Vegf* expression, causing renal epithelial cell necrosis and worsening renal injury. These studies emphasize the essential role of the Hif-1α/Vegf pathway in preserving renal tissue integrity and its disruption as a key factor in diabetic kidney disease progression. Additionally, Yu et al. (2016) [61] showed that hyperglycemia alters gene modulation involving *Hif-1α* and *Vegf*, impacting mitogen-activated protein kinase (MAPK) signaling and brain edema formation, thereby influencing neurological damage in intracerebral hemorrhage.

Thus, the observed alterations are likely driven by the sustained upregulation of *Hif-1α*, reflecting prolonged uncontrolled glycemic dysregulation. This persistent expression can finely regulate downstream targets such as *Vegf*, as demonstrated in both brain and kidney tissues. Similarly, Raimundo et al. (2023) [24] reported comparable changes in renal tissue, linking decreased *Hif-1α* expression to widespread glomerular alterations, which may result from vasoconstriction and renal tubular inflammation mediated by the modulation of *Vegf*.

In parallel with these changes, our immunohistochemical findings show a significant increase in collagen deposition within the kidney. A significant elevation in type I, type III, and total collagen levels was observed, corroborating previous evidence linking excessive collagen accumulation to metabolic stress and the progression of severe renal alterations [60,61,62,63]. Collagen is a major structural component of the glomerular basement membrane (GBM), and its upregulation is considered a compensatory response to endothelial dysfunction commonly observed in diabetic conditions [64,65]. In the diabetic milieu, increased oxidative stress and inflammatory pathways stimulate enhanced collagen synthesis throughout the renal tissue [66,67]. This process is associated with the overexpression of pro-fibrotic genes, leading to mesangial expansion and thickening of the basement membrane, which are hallmark features of progressive renal fibrosis.

Our data suggest that dysregulation of *Hif-1α* expression in both the renal and bulbar regions contributes to systemic hypoxia, which, along with decreased *Vegf* expression, impairs renal angiogenesis and oxygen supply, thereby exacerbating local inflammation and oxidative stress. These deleterious effects disrupt endothelial homeostasis and accelerate renal fibrosis, as evidenced by the significant increase in type I and III collagen deposition observed in our study. The inability to adequately respond to metabolic and hypoxic stress exacerbates microvascular dysfunction, promoting the progressive deterioration of renal and cerebral structures. We hypothesize that this suppression of protective pathways initiates a cascade of cellular injury and programmed cell death, predominantly through apoptotic mechanisms, which was particularly evident during the early stages of diabetes. The observed deactivation of anti-apoptotic responses highlights the complex imbalance in cellular survival signaling caused by chronic glycemic dysregulation.

Recent literature on glucose and lipid metabolism [68] offers an in-depth analysis of the metabolic disturbances that underlie vascular dysfunction, chronic inflammation, and tissue injury—processes central to the pathogenesis of diabetic complications. These systemic metabolic imbalances are particularly detrimental to highly vascularized and metabolically active organs such as the brain and kidney, exacerbating hypoxia-driven signaling pathways including Hif-1α and Vegf. Such interplay between altered metabolism and hypoxia-related molecular mechanisms substantiates and strengthens our findings, highlighting the multifactorial nature of diabetic tissue damage and the pivotal role of these pathways in the progression of complications in target organs.

The observed mortality rate following alloxan administration is in line with reports of increased early mortality due to acute β-cell destruction and systemic oxidative stress [69,70]. In contrast, STZ induces diabetes more gradually, resulting in lower early mortality but differing pathophysiological dynamics [71]. The choice between models should be aligned with the experimental objective. In our study, the rapid onset of metabolic disturbances enabled the investigation of early molecular responses in target tissues.

Although this study did not directly address the role of microbiota or microRNAs, these mechanisms are increasingly recognized in diabetes-related complications. For instance, recent evidence points to the influence of gut microbiota on metabolic inflammation and insulin resistance [72], and to the regulation of renal and neural gene expression by microRNAs [73]. These aspects should be explored in future studies.

This study provides novel insights into the early molecular events of diabetes, emphasizing organ-specific and temporal changes in gene expression related to energy metabolism and hypoxia. Understanding these pathways may improve our comprehension of disease progression and inform the development of targeted therapeutic strategies for diabetic complications. Although our study did not extend to later stages of diabetic complications, the aim was to identify early gene expression shifts that precede structural damage. Prior evidence has shown that early activation of hypoxia-inducible and metabolic genes may reflect initial tissue stress. This is an important initial study to understand the disease mechanisms and their consequences, providing a basis for future investigations in humans, as short-term models are valuable for elucidating the molecular mechanisms that initiate chronic injury.

This study presents some limitations that should be considered when interpreting the findings. One of the main constraints was the induction of diabetes exclusively in male rats, which restricts the generalizability of the results. Future studies should include both sexes to better capture potential sex-related differences in disease progression and molecular mechanisms. Moreover, although this study assessed multiple metabolic and tissue-related parameters at different time points (7, 21, 30, and 40 days) to explore the temporality of disease progression, the inclusion of a broader range of genes—particularly those linked to cellular energy metabolism—would provide deeper insights. A longitudinal approach, extending from early to later stages of diabetes, could further elucidate the molecular dynamics underlying disease development.

We also acknowledge the limitations of using a short-term diabetes model in rodents, as it does not fully reproduce the chronicity, comorbidities (e.g., hypertension and dyslipidemia), or the low-grade systemic inflammation typically seen in humans. These aspects have been addressed in the revised Discussion and Limitations sections to better contextualize our findings.

Furthermore, due to ethical and logistical constraints, the number of animals used was reduced in accordance with the 3Rs principle of animal experimentation (Replacement, Reduction, and Refinement). Despite the small sample size, rigorous non-parametric statistical methods (Kruskal–Wallis test) were employed to address data variability and distribution. Nevertheless, future studies involving larger cohorts are essential to confirm the present findings and to explore the identified trends more comprehensively.

## 4. Materials and Methods

### 4.1. Experimental Design

This was an experimental, case-control study that evaluated 68 male Wistar rats (120–180 g) of the species Rattus norvegicus placed in the housing facility of Centro Universitário FMABC. The animals were housed in boxes, with up to four animals per box, under the following conditions: (a) a 12 h light/dark cycle; (b) room temperature of 21 (±2 °C); and (c) ad libitum access to water and rodent chow. All animals were allowed to habituate for at least five days prior to the experiments.

**Scheme 1:**
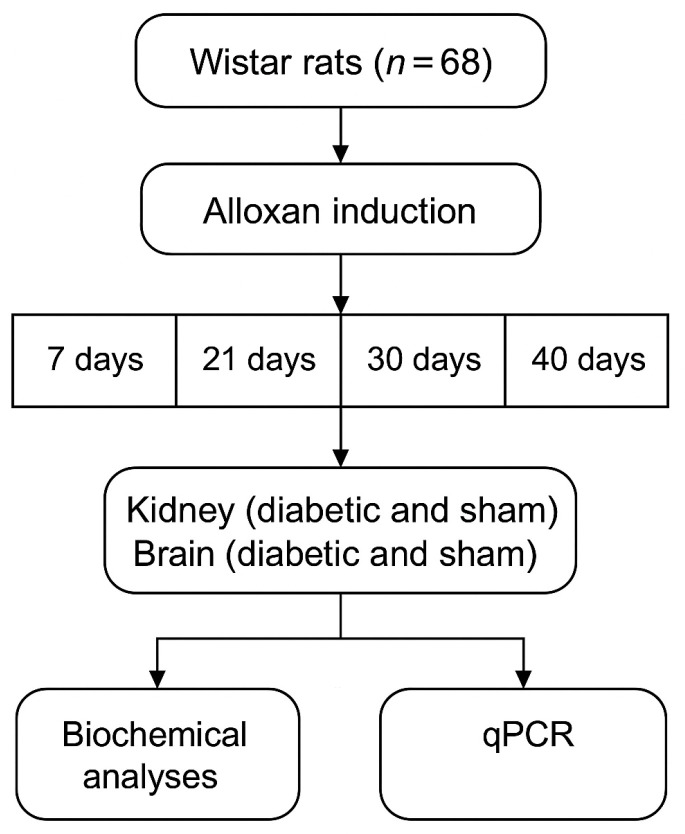
Diagram of DM group animals subjected to intraperitoneal injection of alloxan. Blood glucose was monitored weekly using a commercial glucometer, and euthanasia was performed according to the experimental time points.

The animals were randomly distributed into two experimental groups, Non-diabetic Sham (NDS) and those induced to DM (diabetic group). In order to observe the progressive development of the disease, the animals were subdivided into four subgroups (final n): 7 days (NDS-7, *n* = 11 and DM7, *n* = 13); 21 days (NDS21, *n* = 10 and DM21, *n* = 8); 30 days (NDS30, *n* = 6 and DM30, *n* = 8) and 40 days (NDS40, *n* = 7 and DM40, *n* = 5) (Scheme 1).

### 4.2. Ethics Committee Approval

The experimental procedures were approved by the Ethics Committee on Animal Use (CEUA) of Centro Universitário FMABC, Santo André, Brazil (protocol nº 06/2018). All methods were conducted in strict accordance with National and institutional guidelines for the care and use of laboratory animals, including the Manual of Good Laboratory Practices and CEUA regulations. This study is reported in compliance with the ARRIVE guidelines (Supplementary Materials). All procedures involving Wistar rats adhered strictly to ethical standards and were continuously monitored by a dedicated veterinary team throughout the entire experiment. A pilot phase was conducted to assess protocol feasibility and evaluate potential mortality risks. Humane endpoints were rigorously observed; any signs of discomfort, pain, suffering, or distress reported to the attending veterinarian resulted in immediate intervention, including humane euthanasia when necessary. Animals were monitored daily by trained personnel, and supportive care measures—such as fluid therapy, temperature regulation, and nutritional supplementation—were consistently applied to minimize suffering. The observed mortality rate of 21% occurred predominantly within the first 48 h after alloxan administration, consistent with the acute oxidative stress profile characteristic of this induction model.

### 4.3. Experimental Induction of Groups

Following the habituation period, all animals were subjected to a 12 h fasting period prior to the initiation of experimental procedures, in accordance with the protocol described by Sheriff et al. (2019) [22]. To induce diabetes (DM), animals received a single intraperitoneal (i.p.) injection of alloxan monohydrate [2,4,5,6-tetraoxypyrimidine; 2,4,5,6-pyrimidinetetron] (Sigma-Aldrich^®^, St. Louis, MO, USA) at a dose of 120 mg/kg, diluted in 0.9% sodium chloride solution. Animals in the non-diabetic sham (NDS) group received equivalent i.p. injections of 0.9% sodium chloride alone. Alloxan is widely used in experimental diabetes research due to its selective cytotoxicity to pancreatic β-cells, mediated by the generation of reactive oxygen species. This targeted β-cell destruction compromises insulin secretion and disrupts glycemic homeostasis in a dose-dependent manner. Alloxan remains a cost-effective and reproducible diabetogenic agent, with consistent results reported across studies, and has previously been validated under similar conditions in our institution. Although streptozotocin (STZ) is more commonly employed in recent protocols, several contemporary studies continue to support the relevance of alloxan for modeling diabetes in rodents [69,70,71,72,73].

To ensure safety and refine the induction parameters, a pilot study was conducted to calibrate the alloxan dose and assess early mortality risk. This preliminary phase informed adjustments to the induction protocol implemented in the present study. Seven days after alloxan administration, blood glucose levels were measured using a commercial glucometer (Accu-Chek Advantage^®^, Roche Diagnostics, Indianapolis, IN, USA) via caudal vein puncture. Animals presenting glycemia ≥250 mg/dL were considered diabetic and were included in the experimental follow-up. These animals were monitored without therapeutic intervention for 7, 21, 30, or 40 days post-induction. Throughout the study, body weight and glycemic values were assessed weekly to characterize the metabolic progression associated with the diabetic condition.

### 4.4. Sample Collections

At the end of the experimental period for each group, as determined in this research, animals from the different experimental groups were euthanized with Thiopental (100 mg/kg, i.p.). Immediately after administration, with the animal still alive, blood samples were collected in a dry tube for serum separation and determination of biochemical parameters. The left kidney was extracted and fixed in formalin for histochemical analyses. In addition, the right kidney and brainstem bulbar region were collected to study the expression of genes of interest.

### 4.5. Determination of Biochemical Parameters

#### 4.5.1. Glycemia

Blood glucose was determined weekly using the photometric method and confirmed using fluoridated plasma by the automated enzymatic-colorimetric method (BioSystems^®^ Glucose Ref. 12503, Barcelona, Spain). The evaluation of this parameter was extremely important for confirming the diabetes model; values ˃250 mg/dL were considered abnormal.

#### 4.5.2. Serum Creatinine

Biochemical analyses of serum creatinine were determined using the kinetic-colorimetric Jaffé alkaline picric method, Creatinine Jaffe Elitech Cat. nº CRCO-0600 (Elitech Group Clinical Systems^®^, Sées, France), according to the manufacturer’s protocol, in a COBAS 8000 (Roche Diagnostics^®^, Indianapolis, IN, USA) device at an absorbance of 510 nm.

#### 4.5.3. Plasma Urea

Urea was determined by the enzymatic/colorimetric method using the Urea UV SL Elitech kit catalogue number URSL-0500 (Elitech Group Clinical Systems^®^, Sées, France) according to the manufacturer’s protocol, in a COBAS 8000 (Roche Diagnostics^®^, Indianapolis, IN, USA) device at an absorbance of 600 nm.

### 4.6. Tissue Extraction for Molecular Biology Analysis

Right kidney and bulbar region tissues were collected in cryovials containing 1% PBS and immediately frozen at −80 °C. Subsequently, the tissues were macerated in buffer solution with TissueRuptor II Cat. No./ID: 990890 equipment (Qiagen^®^, Hilden, Germany) and 300 μL (50–100 mg) were added to 1000 μL of TRIzol and the protocol for RNA extraction was followed. Total RNA concentration and the 260/280 ratio were estimated through spectrophotometric reading in the NanoDrop Lite equipment (GE Health Care^®^, Chicago, IL, USA).

### 4.7. Synthesis of DNA Complementary to Messenger RNA (cDNA)

RNA samples were diluted to a concentration of 1 μg and cDNA synthesis was performed with the QuantiTect Reverse Transcription kit Cat No./ID: 205313 (Qiagen^®^, Hilden, Germany), according to the manufacturer’s protocol.

### 4.8. q-PCR in Gene Expression

Expression of the Mct4, Vegfa, Cd147 and Hif-1α genes was evaluated by real-time PCR (qPCR). To normalize the expression values of the target genes, glyceraldehyde-3-phosphate dehydrogenase (Gapdh) expression was used as a reference gene. The specific primers for each selected gene were designed using Primer3 Input 0.4.0 software, available at http://primer3.ut.ee, accessed on 20 May 2022. The designed primer sequences were checked for specificity using the Primer-BLAST program, available at http://www.ncbi.nlm.nih.gov/tools/primer-blast, accessed on 20 May 2022.

The primers used for gene expression analysis by quantitative PCR were designed to amplify specific regions of the target genes, generating amplicons of defined lengths. For *Gapdh* (housekeeping gene), producing a 102 bp amplicon. For *Mct4*, 150 bp amplicon. The *Cd147* gene 169 bp product. *Hif-1α* 195 bp amplicon. *Vegf* 130 bp (The primer designs contained in this article are under patent protection).

The real-time amplification reactions were performed with an Applied Biosystems 7500 Real Time PCR Systems thermal cycler (Applied Biosystems^®^, Waltham, MA, USA), in a final reaction volume of 15 μL and containing: 1× SYBR Green Mix (Quantitec SYBR Green PCR Cat kit No./ID: 204343, Qiagen, Hilden, Germany), 1.5 μL of cDNA and 10 pmol of each specific primer in the following concentrations: Gapdh at 0.2 µMol and Mct4, Vegfa, Cd147 and Hif-1α at 0.15 μMol. The thermal profile was determined with an initial heating step at 95 °C for 10 min, followed by 45 repetitions at 95 °C for 15 s and at 60 °C for 25 s.

The calibration curve of each gene was performed with serial dilutions (1, 1:10 and 1:100) of cDNA synthesized from a concentration of 1 μg of total RNA obtained from tissues and control leukocytes of normal animals. Gene expression was calculated by applying the 2 (−ΔCq) formula and its results presented as expression difference followed by interval (minimum and maximum) [23,24].

### 4.9. Picrosirius

The left kidney was sectioned into 5 μm slices using a microtome. The histological sections of the wound were stained using the Picrosirius method. After deparaffinization and hydration, the sections were stained for one hour in a 0.1% Sirius Red solution, dissolved in saturated aqueous picric acid, ensuring complete coverage of the tissue sections. The slides were then washed in running water for three minutes, counterstained with Carazzi’s hematoxylin for four minutes, and washed again in running water for five minutes before being dried for subsequent photographic analysis. Morphological analyses of renal tissue were conducted using light microscopy (Nikon Eclipse E200 microscope at 100× magnification, Nikon Corporation^®^, Tokyo, Japan). For each animal, ten images of the kidney were acquired per section in a zigzag pattern to ensure representative sampling of the tissue. The analysis focused on the identification and quantification of type I and type III collagen fibers. Final results were expressed as the mean collagen content calculated from the ten analyzed fields per animal.

### 4.10. Statistical Analyzes

Results are presented as mean and standard deviation (SD) with a 95% confidence interval (5% significance level). Quantitative variables were compared with unpaired Student’s *t*-test for parametric values (mean blood glucose and plasma biochemical parameters) and the Mann–Whitney test for non-parametric values (gene expression), with the distribution evaluation analyzed by Shapiro–Wilk (*p* > 0.05). For analysis of the temporal correlation between the studied genes and the experimental subgroups, the one-way ANOVA test was performed. The program used for analysis was GraphPad Prism (GraphPad^®^, version 7.0, San Diego, CA, USA).

## 5. Conclusions

In conclusion, this study highlights the early, time-dependent dysregulation of key metabolic and hypoxia-related genes—namely *Mct1*, *Mct4*, *Hif-1α*, and *Vegf*—in both brain and kidney tissues of diabetic rats. The downregulation of *Mct1* and *Mct4* suggests impaired monocarboxylate transport and altered energy metabolism, particularly within the brainstem, potentially contributing to early neuronal dysfunction. Simultaneously, the decreased expression of *Hif-1α* and *Vegf* may reflect compromised hypoxia signaling and angiogenesis, associated with microvascular rarefaction and the initial stages of renal fibrosis. These molecular changes were accompanied by the increased deposition of type I and III collagen in the kidney, indicating early extracellular matrix remodeling and fibrotic activation. Notably, these alterations occurred in the absence of overt histological damage, suggesting that gene expression changes may precede detectable structural abnormalities.

Although the magnitude of the observed effects was modest, the consistency and statistical significance support the relevance of these early molecular shifts as potential biomarkers of tissue stress and dysfunction in diabetes. Our findings underscore the intricate interplay between metabolic, vascular, and fibrotic mechanisms in the early stages of diabetic complications. Given the preliminary nature of this study, further research is warranted to validate these molecular markers, explore their mechanistic roles, and evaluate their potential as early therapeutic targets to prevent or attenuate diabetes-related organ damage.

## 6. Future Perspectives

Future studies should focus on longitudinal designs with extended follow-up periods to better capture the progressive nature of diabetes-induced damage in renal and neural tissues. Expanding the scope of molecular analyses beyond gene expression to include circulating microRNAs and/or inflammatory mediators such as interleukins will provide a more comprehensive understanding of the underlying pathophysiological mechanisms. Furthermore, the evaluation of potential therapeutic interventions—particularly antioxidant and anti-inflammatory compounds—could offer insights into strategies to mitigate early tissue damage and improve clinical outcomes.

## Figures and Tables

**Table 1:** Analysis of plasmatic biochemical parameters between the NDS and DM groups through progressive analysis in the different sample time groups (7, 21, 30 and 40 days). Mann–Whitney and Student’s *t*-test (95% confidence interval: 95%CI) * *p*  <  0.05 vs. NDS. Abbreviations: Non-Diabetic Sham (NDS); Diabetic group (DM) and Standard Deviation (SD).

Variables	Times	NDS Average (±SD)	DM Average (±SD) (*n* = 13)	*p* Value
Plasma glucose (mg/dL)	7 days	190.63 (±56.78)	599.58 (±139.29) *	<0.0001
	21 days	181.33 (±34.70)	777.20 (±233.74) *	<0.0001
	30 days	169.18 (±32.09)	763.74 (±221.40)*	<0.0001
	40 days	195.50 (±38.02)	763.28 (±77.92) *	<0.0007
Plasma creatinine (mg/dL)	7 days	0.42 (±0.06)	0.80 (±1.24) *	0.04
	21 days	0.46 (±0)	1.99 (±4.02)	0.41
	30 days	0.86 (±0.12)	1.23 (±0.45) *	0.03
	40 days	1.13 (±0.42)	1.72 (±0.73)	0.18
Plasma urea (mg/dL)	7 days	57.43 (±14.71)	97.94 (±28.27) *	<0.0002
	21 days	68.31 (±5.08)	153.38 (±44.13) *	<0.0001
	30 days	79.62 (±7.15)	170.20 (±21.00) *	<0.0007
	40 days	82.52 (±12.00)	173.10 (±42.71) *	<0.01

## Data Availability

The datasets generated and/or analysed during the current study are available in the ArrayExpress repository (E-MTAB-13080) from this link: <https://www.ebi.ac.uk/biostudies/arrayexpress/studies/E-MTAB-13080?key=ae3eb876-00b6-4899-a332-07665d6e316a> (accessed on 16 June 2023).

## Supplementary Materials

The supporting information can be downloaded at: https://www.mdpi.com/article/10.3390/ijms26199676/s1.

## Abbreviations

The following abbreviations are used in this manuscript:

Abbreviation	Full Term
AMPK	AMP-activated Protein Kinase
CD147	Cluster of Differentiation 147
CEUA	Ethics Committee on Animal Use
CNS	Central Nervous System
DKD	Diabetic Kidney Disease
DM	Diabetic group
EMMPRIN	Extracellular Matrix Metalloproteinase Inducer
GAPDH	Glyceraldehyde-3-phosphate Dehydrogenase
GBM	Glomerular Basement Membrane
HIF	Hypoxia-Inducible Factor
MAPK	Mitogen-Activated Protein Kinase
MCT	Monocarboxylate Transporters
MMP	Matrix Metalloproteinases
mTOR	Mechanistic Target of Rapamycin
NDS	Non-Diabetic Sham
PHD	Prolyl Hydroxylase Domain
RAAS	Renin-Angiotensin-Aldosterone System
ROS	Reactive Oxygen Species
RVLM	Rostral Ventrolateral Medulla
SD	Standard Deviation
STZ	Streptozotocin
VEGF	Vascular Endothelial Growth Factor
