# Supplementary material for: Short-Term: Cellular Metabolism and Gene Expression During the Onset of Diabetic Kidney Disease: A Diabetes Mellitus Experimental Model
Source: Int J Mol Sci. 2025 Oct 4;26(19):9676. doi: 10.3390/ijms26199676 (PMC12524569; doi:10.3390/ijms26199676)
Supplement: Supplementary file 1 [file ijms-26-09676-s001.zip › Mortality_graph.pdf]

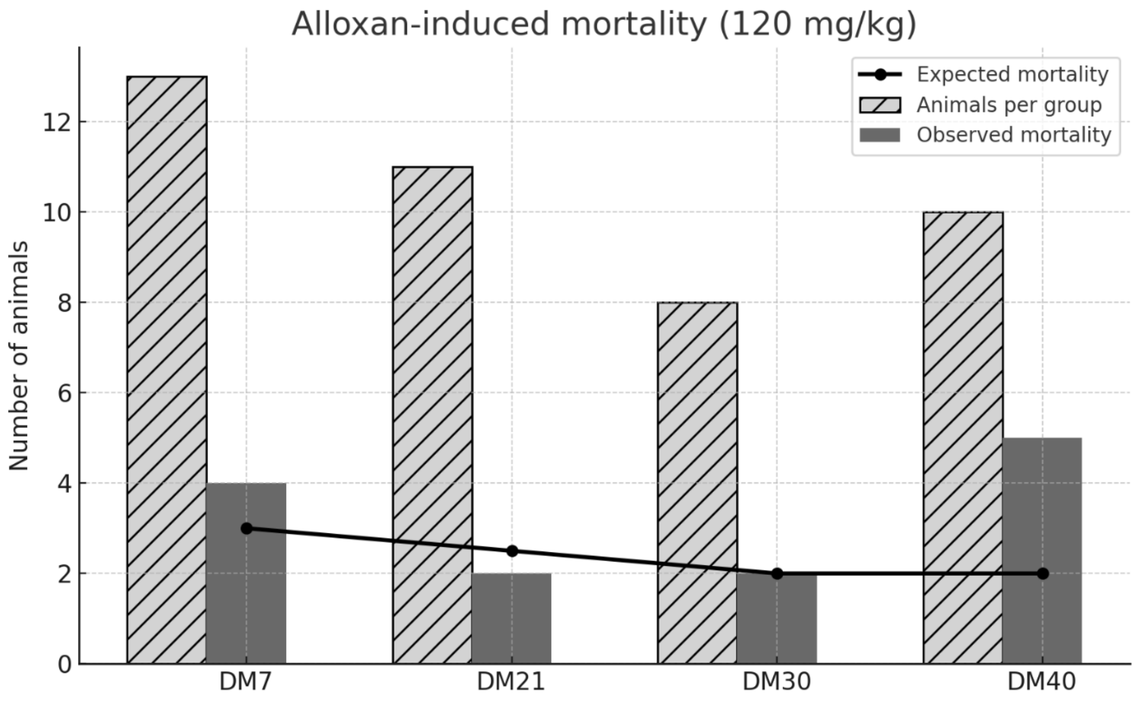

Figure 1: Expected versus observed mortality in Wistar rats after alloxan-induced diabetes (120 mg/kg). Each bar represents the number of animals per group (n = 13). The gray bars indicate the observed mortality, while the dashed line shows the expected mortality (approximately 20–30% based on pilot studies and literature). The discrepancy highlights that the main cause of mortality was cachexia, consistent with diabetic progression.
