# Supplementary material for: Short-Term: Cellular Metabolism and Gene Expression During the Onset of Diabetic Kidney Disease: A Diabetes Mellitus Experimental Model
Source: Int J Mol Sci. 2025 Oct 4;26(19):9676. doi: 10.3390/ijms26199676 (PMC12524569; doi:10.3390/ijms26199676)
Supplement: Supplementary file 1 [file ijms-26-09676-s001.zip › Supplementary file_Cq_GAPDH.pdf]

Ct GAPDH - DM heart	Ct GAPDH - NDS heart	Ct GAPDH - DM brain	Ct GAPDH - NDS brain
29,422	26,567	26,799	25,142
4,371	4,875	4,740	5,240

Ct GAPDH - DM kidney	Ct GAPDH - NDS kidney	Ct GAPDH - DM blood	Ct GAPDH - NDS blood
27,268	24,466	29,310	21,206
5,497	7,499	5,168	6,425

Ct GAPDH - DM liver	Ct GAPDH - NDS liver
22,209	27,608
2,407	4,830

Ct GAPDH - DM urine	Ct GAPDH - NDS urine
32,657	30,671
2,802	3,141
