# Supplementary material for: Short-Term: Cellular Metabolism and Gene Expression During the Onset of Diabetic Kidney Disease: A Diabetes Mellitus Experimental Model
Source: Int J Mol Sci. 2025 Oct 4;26(19):9676. doi: 10.3390/ijms26199676 (PMC12524569; doi:10.3390/ijms26199676)
Supplement: Supplementary file 1 [file ijms-26-09676-s001.zip › Table_Ct_values.pdf]

GRUPOS	Ct GAPDH	Ct MCT1	Ct MCT4	Ct CD147	Ct HIF1a	Ct VEGF
DM7-1	23,76505661	26,81727028	32,30796814	26,5128479	33,63755035	26,88387299
DM7-2	21,37158585	25,36913681	30,34992981	24,69682312	29,64391327	19,64676285
DM7-3	29,96827698	33,40957642	33,80756378	32,18971252	36,70901108	24,74126625
DM7-4	33,04094315	35,47649384	NE	34,09887695	32,17259216	30,23957825
DM7-5	32,2816391	32,38697815	NE	31,28770447	35,36666489	23,96137238
DM7-6	32,31147003	33,63140869	33,08232117	32,78465271	35,99230194	27,44987106
DM7-7	32,50046539	36,4056282	NE	36,0524826	40,14680099	28,20796204
DM7-8	29,53166199	30,75138474	34,15018845	29,17916489	31,24795914	
DM7-9	32,49245453	35,08055115	NE	33,31224823	37,74121094	
DM7-10	32,45470428	37,20524216	NE	36,60220337	40,79508209	30,40726089
DM7-11	32,50941467	35,09412766	33,63556671	33,20793915	37,63774872	
DM7-12	21,38476181	25,32606506	27,49036217	21,66776657	25,75997162	17,73884964
DM7-13	28,87676239	25,16889572	27,23413658	24,63191223	25,13619232	22,90222931
NDS7-1	22,38684464	26,57030869	32,43223572	25,33955383	31,56796646	
NDS7-2	22,83737564	26,85135269	32,44258118	24,5248642	31,05979156	
NDS7-3	32,90129089	35,28462982	NE	33,43158722	38,5279808	29,86992645
NDS7-4	29,56055832	33,05636215	32,1443634	30,2101078	35,68277359	
NDS7-5	34,25856018	27,64741135	NE	NE	NE	NE
NDS7-6	30,19922638	34,30108643	NE	32,8899765	36,72833252	
NDS7-7	30,46264267	26,60150909	28,51544952	25,39902306	26,02573395	23,34908485
NDS7-8	22,01622772	25,2759819	27,87661743	26,03709221	25,29798508	24,44482803
NDS7-9	22,24043274	26,8952446	27,92563248	27,22816658	28,33563614	25,70545578
NDS7-10	22,93229294	26,22517395	27,70233536	26,25620651	27,54100418	21,47932625
NDS7-11	22,43924141	26,99822617	26,87324905	26,41057587	27,54496193	25,26196671
DM7-1	19,77244186	24,26470947	28,19106293	21,79507637	28,08195686	21,03976059
DM7-2	21,08512497	27,71800613	26,98341751	23,19311905	28,69000626	24,07625961
DM7-3	21,57288361	29,18426514	31,46095276	25,34584427	31,59248352	
DM7-4	26,7644043	25,41113281	27,98265076	22,23879623	26,00205612	23,5574894
DM7-5	29,09001541	28,03883743	29,70743561	25,14348602	28,98428917	22,84179497
DM7-6	27,90720367	29,03058243	30,48794746	25,14612579	30,08188629	24,10141182
DM7-7	30,77960777	27,07527542	28,57196426	24,45724487	29,02135658	22,58516693

Coração

NE: did not express

Cérebro	**DM7-8**	29,66588402	26,54038048	25,84951782	23,74762726	28,43018341	23,19102859
	**DM7-9**	29,02066422	25,83675003	28,07332993	23,55654907	26,45847702	
	**DM7-10**	32,92869568	30,32331467	29,22324562	28,61943054	30,24487495	27,2281456
	**DM7-11**	30,92093658	28,57171631	30,64088058	25,81569099	29,85396194	23,3700676
	**DM7-12**	29,87428474	23,51870155	27,53133774	18,2443161	18,22555733	20,34754562
	**DM7-13**	19,00321198	23,33418465	23,37992096	17,31309128	20,00466156	17,97826004
	NDS7-1	20,44952011	26,20659065	30,00057411	23,31586838	28,28396225	22,51267624
	NDS7-2	20,30000877	25,88941193	30,14328766	22,82177734	28,28750992	21,3196373
	NDS7-3	26,21949005	29,03289413	28,34882736	24,39443207	29,71968269	23,37624359
	NDS7-4	29,61205292	26,65280914	24,90245438	22,87570763	28,25888062	
	NDS7-5	32,48316956	30,11755562	32,11821747	26,54792976	31,77899551	22,93800354
	NDS7-6	28,36689377	28,24420547	30,0488987	27,29494667	30,01481056	
	NDS7-7	29,62755203	23,22525406	26,58244324	16,7609024	18,12740707	20,00373459
	NDS7-8	30,62922668	23,90187073	27,22634125	18,25168991	18,94032669	20,72949982
	NDS7-9	19,84636688	23,91732788	27,86750412	18,98247337	22,0145359	20,63220215
	NDS7-10	18,51263428	21,76226234	26,5238266	16,12112999	16,75923157	19,94497299
	NDS7-11	20,50983047	25,45922089	28,16978455	17,81150055	22,92308807	18,64827919
Fígado	**DM7-1**	20,60079575	26,39554977	32,25987244	23,8879509	29,53070831	21,25042343
	**DM7-2**	20,52039909	25,92414093	30,52866745	23,07124138	29,56004524	16,70120239
	**DM7-3**	15,25343513	29,90090179	31,38843155	7,79337883	15,00986481	22,73595047
	**DM7-4**	24,08279037	27,8957901	27,09947395	23,90717316	28,89744186	19,58947563
	**DM7-5**	22,93813705	27,5711441	30,0712204	22,00868225	28,0995121	18,52235031
	**DM7-6**	23,82785988	26,54572296	27,87485123	22,91993713	26,82300949	18,92778015
	**DM7-7**	23,97305679	26,90755272	27,57021713	23,00958633	27,83825684	18,55596161
	**DM7-8**	22,35755539	26,32985878	30,74477768	21,7328434	28,25206375	19,35274506
	**DM7-9**	22,87797165	26,64848137	**NE**	23,89056396	27,51058006	20,24550247
	**DM7-10**	22,20605659	25,25928688	30,28224945	19,44641495	25,58426666	17,50764275
	**DM7-11**	24,15450859	28,8516922	27,36151886	25,5729332	29,02952957	21,28430367
	**DM7-12**	22,43552399	24,88092232	29,41940689	19,26020622	25,73303986	16,97294998
	**DM7-13**	23,49423981	25,86927032	**NE**	23,99554825	24,72916794	20,13103867
	NDS7-1	28,41088104	28,50661087	32,72729111	25,07779503	31,02518463	
	NDS7-2	22,44011307	26,43079758	31,76405716	23,69245148	28,95785522	

	NDS7-3	32,5779953	32,89089966	33,04005432	33,31131744	38,60545731	
	NDS7-4	30,34173584	34,53779602	**NE**	34,40611267	38,05685425	
	NDS7-5	22,27332306	24,75839996	27,19831467	19,64875984	24,11141968	
	NDS7-6	20,48411179	17,0786972	22,51016998	7,109222889	16,06483078	
	NDS7-7	31,01416397	21,93897247	29,95620346	19,70580673	21,50408363	
	NDS7-8	32,23145294	25,5423851	30,33278465	23,83504486	22,03064346	
	NDS7-9	24,53649712	26,38647079	**NE**	23,64035988	27,54806137	
	NDS7-10	25,02552223	28,26745224	**NE**	25,2937355	29,33647156	
	NDS7-11	34,34967422	26,98931122	30,83216667	25,61364174	25,86336517	
	**DM7-1**	21,33720398	27,36875725	27,68294144	22,45132065	29,5759201	16,58525085
	**DM7-2**	22,11645508	28,25175667	30,19435692	24,19682312	31,08993149	
	**DM7-3**	19,45772171	35,04767609	32,19074249	29,36118317	33,27377319	21,13835526
	**DM7-4**	22,35843849	34,90629959	30,58959198	28,04449081	32,03372192	23,63744736
	**DM7-5**	29,34385109	20,92527008	33,57129669	24,29725838	24,53422546	25,01190186
	**DM7-6**	32,47657394	34,68247223	32,56279755	29,34416008	34,07779694	18,24082947
	**DM7-7**	32,2490387	36,81033707	31,52376938	29,83089447	32,93651962	20,94802094
	**DM7-8**	32,78339767	34,03312302	31,02070999	28,26862717	32,24459839	20,8296051
	**DM7-9**	32,570858	**NE**	36,87237167	21,08083916	18,24393845	22,7486496
**Rim**	**DM7-10**	32,72531128	35,06938934	34,70103455	22,61062241	15,0953474	
	**DM7-11**	32,16102982	29,3795929	29,54709625	24,42411804	29,67519379	19,74434853
	**DM7-12**	21,81830978	28,32212067	27,92144775	22,90416336	24,59849739	23,25458908
	**DM7-13**	23,07978439	29,72584343	25,40088272	20,09643555	24,27905655	20,47382736
	NDS7-1	20,62034416	27,40569496	28,79630852	22,48021317	28,39314461	18,52001572
	NDS7-2	20,76310349	27,44776535	30,33564568	23,12432861	28,92437744	17,40926361
	NDS7-3	31,36952782	37,12521744	**NE**	32,66355133	37,42733765	24,16145515
	NDS7-4	24,75866318	35,75314713	**NE**	33,08701324	36,01515198	23,92434311
	NDS7-5	32,62751389	35,74484253	33,33239746	29,57879257	32,66053772	17,41008949
	NDS7-6	32,35704041	34,85836029	**NE**	**NE**	13,9119873	
	NDS7-7	22,46664619	28,67247772	26,04609489	26,88479233	26,85160065	24,91905975
	NDS7-8	23,82447433	30,64344788	26,617733	25,94486046	28,18750572	24,25263977
	NDS7-9	24,53649712	27,40648079	28,13749313	23,81157875	22,89690399	22,54559326
	NDS7-10	29,44798279	27,60531235	27,75066376	23,56149483	22,83862877	22,2104435

Sangue

NDS7-11	6,355296135	6,726030827	18,42219162	12,03622818	22,05077362	20,7503109
DM7-1	20,84486389	26,81696701	23,96824646	26,16578484	29,02780151	
DM7-2	21,21495438	27,40309525	24,45048332	26,38090134	28,42346954	
DM7-3	32,5206604	28,6240387	27,91676712	27,6126461	30,14710999	
DM7-4	32,09809875	29,23223114	27,93627167	28,33196259	30,49120903	
DM7-5	31,55322647	28,57225037	28,25476837	29,81400299	30,85069466	
DM7-6	33,00650787	30,80024529	31,60562134	30,51152802	31,59509087	
DM7-7	31,07185364	29,89554214	30,47052956	30,61077499	32,86894608	
DM7-8	31,14472771	28,96470642	29,25191879	30,26737595	31,34796906	
DM7-9	31,18865204	30,38990784	27,55582809	29,41511154	30,5305481	
DM7-10	31,28569031	30,66477203	28,83860588	29,46862411	30,38473129	
DM7-11	29,42276764	26,51794052	25,10437012	26,07775879	28,29379272	
DM7-12	35,84395599	NE	NE	36,861248	NE	
DM7-13	19,82843018	25,47205353	19,33903313	20,58058929	21,46784592	
NDS7-1	20,13437271	28,09563828	24,18950653	26,72449875	28,92608643	
NDS7-2	21,06447983	26,68358803	24,19452667	25,50277328	27,79370117	
NDS7-3	19,8602581	27,51954079	25,69019127	25,51312256	28,74900055	
NDS7-4	19,95302963	26,86486244	24,62252426	24,81846619	26,97395897	
NDS7-5	31,17714882	30,1561985	NE	28,04188919	30,63282013	
NDS7-6	24,33782387	31,02950478	NE	30,70892715	33,67987061	
NDS7-7	18,80161667	25,14548492	20,6236763	22,44358063	22,62929344	
NDS7-8	31,00737381	NE	NE	NE	NE	
NDS7-9	17,69431686	23,48342133	18,64764786	20,66283035	20,66012383	
NDS7-10	7,614658356	24,28883362	22,65296745	7,79716301	9,784896851	
NDS7-11	21,61577797	27,81983948	26,81833458	25,61810112	26,38403702	
DM7-1	33,56267548	NE	NE	37,1084137	39,76970291	
DM7-2	33,61616516	NE	NE	39,85832977	NE	
DM7-3	32,08669281	38,15448761	NE	34,98908997	38,6059494	
DM7-4	32,73247528	NE	NE	38,66318512	NE	
DM7-5	30,89980316	28,03315353	27,38062477	29,19126129	30,59300232	
DM7-6	33,93476868	NE	NE	38,0480423	39,94371033	
DM7-7	33,96066666	NE	NE	NE	NE	

Urina	DM7-8	33,96066666	NE	NE	37,92362976	36,95177841
	DM7-9	34,48516083	NE	NE	NE	21,4085598
	DM7-10	34,49549103	NE	NE	37,02991867	NE
	DM7-11	25,57702637	28,12591934	28,21915817	23,15110397	26,92590714
	DM7-12	36,30363083	33,15501785	NE	NE	NE
	DM7-13	28,92780495	NE	NE	NE	NE
	NDS7-1	34,9283905	37,50379562	NE	NE	NE
	NDS7-2	33,90021515	NE	NE	NE	NE
	NDS7-3					
	NDS7-4	33,19689178	NE	NE	NE	NE
	NDS7-5	32,77401352	NE	NE	36,06955719	38,1452179
	NDS7-6	32,40229416	38,27279663	35,59014893	36,28825378	38,53529358
	NDS7-7	26,55145645	33,83936691	NE	29,93793488	30,7424984
	NDS7-8	29,84454155	36,93078232	NE	33,09927368	NE
	NDS7-9	27,90000153	33,54265594	NE	19,66638756	21,58913612
	NDS7-10	26,51981354	34,67154694	28,56337166	30,15063858	31,61590195
	NDS7-11	28,69549179	36,58990479	NE	37,0358429	37,53622818
Coração	DM21-1	21,06999969	23,86093521	28,0284214	20,30524635	24,83449364
	DM21-2	30,17384529	28,52597809	33,02629852	28,48210526	33,00116348
	DM21-3	31,12344742	31,73658371	35,36379242	29,41646194	32,76600266
	DM21-4	28,20326233	23,47890091	30,67067719	22,94714737	27,77762985
	DM21-5	29,56318283	35,636417	NE	32,3957	NE
	DM21-6	29,97384262	25,70172501	33,29930878	24,98156357	26,50159836
	DM21-7	29,42025375	32,81743622	34,25355911	24,93035889	14,93136597
	NDS21-1	22,29584694	26,728508	27,60154724	26,74034691	27,64736748
	NDS21-2	29,01397324	28,22776413	34,20108032	25,07865715	NE
	NDS21-3	28,86153793	31,27434158	34,92441559	30,37364578	NE
	NDS21-4	34,14207077	36,91290665	NE	32,61643219	37,06033707
	NDS21-5	16,3190155	NE	NE	35,343803	NE
	NDS21-6	30,65628052	34,27615356	33,443	31,139168	31,102837
	NDS21-7	29,3010025	29,43761635	33,00524902	26,077702	29,36147

	NDS21-8	29,450687	35,31725311	36,08400345	29,69521332	29,46097374
	NDS21-9	29,84958649	34,84445	NE	34,410892	36,452557
	NDS21-10	30,87509155	NE	NE	34,889107	NE
	DM21-1	18,81423378	23,86142349	26,99209785	16,54441833	20,97774506
	DM21-2	24,39258575	NE	NE	21,51661301	23,25631142
	DM21-3	26,05716515	30,76002884	32,62388992	21,722572	24,268158
	DM21-4	26,92100525	28,7649498	32,71739197	22,92750549	26,35952377
	DM21-5	25,00489807	21,62037277	30,90416145	19,24466324	23,43056297
	DM21-6	25,02108383	21,49815369	30,03595734	19,41937256	19,94378662
	DM21-7	24,33663368	30,86751366	29,04346085	29,29961	22,752254
	NDS21-1	16,27931213	19,9159584	18,97394562	16,37911224	20,45305061
Cérebro	NDS21-2	27,54353714	28,23749542	32,96396637	23,29508209	27,63262939
	NDS21-3	18,36970329	24,73465919	30,52401543	20,274036	27,465528
	NDS21-4	23,82366562	26,31240845	31,56820107	19,60264015	23,10665131
	NDS21-5	26,61037827	26,20256042	31,89953804	18,42319489	20,95139503
	NDS21-6	25,26070595	NE	28,591	20,159416	27,165081
	NDS21-7	25,98051834	27,91870117	31,40852737	22,862297	25,043964
	NDS21-8	27,3716774	33,492172	33,423687	28,37101	29,84045
	NDS21-9	29,410328	33,95424271	36,83722305	26,0231266	26,05858231
	NDS21-10	28,03081322	33,556633	NE	28,781685	30,492973
	DM21-1	23,10058975	25,18427277	29,26025963	22,00017738	26,0850296
	DM21-2	30,62410736	32,2387085	35,35439301	29,50606537	33,73692322
	DM21-3	28,0730896	26,23016167	29,40281677	21,95232	25,322104
	DM21-4	30,27620506	31,97255325	34,02109528	27,03297043	31,55149651
	DM21-5	25,83369064	20,77943039	32,14228821	19,04353714	24,92542267
	DM21-6	27,82226181	23,36860275	33,04341888	22,88663864	23,81012344
	DM21-7	32,02894211	34,99153137	32,23497772	27,860987	32,109253
	NDS21-1	23,60491943	28,07557678	NE	25,14644432	28,60319519
Fígado	NDS21-2	30,90228271	28,98253059	34,10803223	26,72708511	30,78752708
	NDS21-3	32,95721817	33,46489716	NE	31,23518753	35,18499756
	NDS21-4	29,59688187	27,86064148	34,51786804	23,8373661	28,26688194
	NDS21-5	32,18252945	30,00783157	34,11489105	25,95494652	31,25927353

	NDS21-6	29,71565247	31,63783455	32,893	25,906633	29,816975
	NDS21-7	32,05892181	34,81109619	NE	30,047039	34,721897
	NDS21-8	37,48272324	NE	NE	NE	NE
	NDS21-9	31,696589	NE	NE	29,83813477	25,44343185
	NDS21-10	29,68652534	34,68596	NE	32,45855	NE
	DM21-1	20,83308411	27,95514297	27,05123711	24,87679291	27,42174911
	DM21-2	31,20423508	35,0676651	35,1595459	28,52829361	32,44997406
	DM21-3	30,33068085	33,65014648	34,39974213	23,86244583	28,35627747
	DM21-4	32,866539	35,70653534	36,9622879	27,7308197	29,64582253
	DM21-5	28,72021866	22,75825882	30,98191833	19,38672638	20,79890251
	DM21-6	30,60132217	27,93917465	33,72279358	25,44174957	25,87824631
	DM21-7	26,34996033	28,63027	27,41971588	20,634552	23,546421
	NDS21-1	18,71637726	24,53277588	25,41497803	22,9155159	23,57769775
Rim	NDS21-2	34,22483444	35,73916626	NE	28,154957	29,902008
	NDS21-3	29,41479492	31,92558289	32,02684784	25,89263916	29,11616516
	NDS21-4	30,52773476	32,09672928	33,0587883	22,81895447	26,1362915
	NDS21-5	32,45329285	24,17072678	35,72047806	28,93846703	28,14692497
	NDS21-6	32,37594223	NE	33,01031494	29,531502	29,544127
	NDS21-7	28,3356514	32,1326828	31,63987732	23,049435	26,759712
	NDS21-8	29,20086479	35,844505	34,62398	32,5839	35,427593
	NDS21-9	34,332558	NE	NE	31,52888107	33,04627228
	NDS21-10	31,94345093	NE	NE	35,264114	NE
	DM21-1	18,33874512	25,11348343	24,1224823	23,60338402	24,02735329
	DM21-2	27,7186451	29,29463196	28,64115906	30,67397118	27,52347183
	DM21-3	27,51330948	27,9174366	27,27889633	25,81005478	25,9434433
	DM21-4	29,1283474	28,47421265	27,67593384	25,09257126	25,16778946
	DM21-5	36,1995697	25,16232681	NE	23,8425827	20,03286934
	DM21-6	29,6854744	24,70984268	26,85737801	24,55794334	22,16755295
	DM21-7	27,60551071	32,44090652	25,29075623	24,35286	24,579054
	NDS21-1	19,83421516	24,72737503	22,1586113	20,51914597	21,60509109
Sangue	NDS21-2	25,77861977	24,75337601	31,0819397	25,41468811	24,28547668
	NDS21-3	27,02440262	27,75073624	25,32180405	25,096241	25,71108627

Urina

NDS21-4	25,84886169	27,39159393	26,05589867	23,60884857	23,56704903
NDS21-5	24,96602821	23,01158524	27,78369522	23,46211815	20,98710251
NDS21-6	28,42808533	30,86491585	29,50514412	26,413876	26,43043
NDS21-7	28,32619095	29,89478302	28,57395172	26,509298	26,44085
NDS21-8	29,51124191	NE	31,882221	33,66874	34,330215
NDS21-9	30,801228	36,08979034	35,01618958	29,85432053	27,17897224
NDS21-10	26,70885468	35,29455	NE	31,700148	33,258186
DM21-1	27,41892624	35,20233917	NE	31,9098835	33,85421371
DM21-2	35,33369064	NE	NE	36,96581268	37,5242691
DM21-3	22,97258186	26,43480301	27,21423721	34,96172714	33,559246
DM21-4	21,38331604	NE	NE	33,75465012	36,66030121
DM21-5	34,93973923	26,73904991	33,66636276	30,79587555	29,65433502
DM21-6	34,37010193	28,19774055	34,30830002	32,96118164	30,75716019
DM21-7	35,86568069	NE	NE	33,329166	28,691936
NDS21-1	28,33804893	35,66145325	NE	NE	38,60563278
NDS21-2	21,52993393	NE	NE	34,38115	28,92430878
NDS21-3	35,41551208	NE	33,244	34,07178	34,52462
NDS21-4	37,23052979	26,44449234	34,77039337	31,93284798	29,5352459
NDS21-5	35,66954803	24,64700127	NE	32,82324982	31,06652069
NDS21-6	34,74770737	NE	35,28029	38,00000	NE
NDS21-7	16,3614006	NE	NE	35,94851	NE
NDS21-8	28,59454346	35,894783	35,036728	35,539734	34,636566
NDS21-9	33,877007	NE	NE	NE	29,66833878
NDS21-10	34,408604	NE	NE	NE	NE

ação

DM30-1	32,250313	36,51655579	NE	28,98535919	NE
DM30-2	34,929596	NE	NE	NE	NE
DM30-3	33,72804	NE	NE	30,7865448	31,46195602
DM30-4	29,471424	35,91347504	NE	30,0550766	29,79442024
DM30-5	34,282566	NE	NE	31,893507	NE
DM30-6	28,979454	31,44136429	36,05182648	25,19936562	18,43821716
DM30-7	29,04007	32,78046799	32,57950974	33,31194687	33,3441124

Cora	DM30-8	31,70681	36,23988342	35,28786087	NE	35,72761536
	NDS30-1	30,657505	NE	NE	33,81128693	36,34503937
	NDS30-2	35,133408	NE	NE	33,49327087	30,73137856
	NDS30-3	33,797348	NE	NE	33,00349808	30,21866226
	NDS30-4	33,226852	NE	36,95930862	32,08263016	30,36470222
	NDS30-5	29,87667	35,58769989	36,62941742	30,21697235	21,45710182
	NDS30-6	35,77433	NE	NE	NE	NE
Cérebro	DM30-1	32,3138237	NE	33,28819	34,786312	33,251396
	DM30-2	31,7218647	NE	32,528233	36,450775	NE
	DM30-3	31,10419464	36,322155	33,606915	NE	33,8432
	DM30-4	30,766068	31,63138008	34,08972549	28,02075577	32,21405029
	DM30-5	30,23945808	36,26502609	30,614037	NE	32,179047
	DM30-6	30,0508194	36,230095	32,578644	33,683643	31,539131
	DM30-7	32,80251312	37,60612	32,85277	33,67549	NE
	DM30-8	27,71977425	29,50866699	33,27249146	25,28186798	29,38716125
	NDS30-1	30,98884201	35,81086	33,95633	35,553204	32,358276
	NDS30-2	31,9632225	34,02869	34,481567	NE	31,693855
	NDS30-3	32,64735031	NE	33,960846	32,9366	33,145195
	NDS30-4	31,91092682	35,64889	36,23408	34,001804	32,842227
	NDS30-5	30,04316711	NE	34,336178	31,948616	33,068184
	NDS30-6	31,9392128	NE	34,70971	34,228245	33,91531
Fígado	DM30-1	28,561028	33,80693817	NE	27,66256714	33,5459404
	DM30-2	30,159023	29,36540604	NE	26,32420731	32,18205643
	DM30-3	38,79171	NE	NE	32,78495789	NE
	DM30-4	33,185036	NE	NE	28,12275887	32,49047089
	DM30-5	32,90207	NE	NE	26,50693893	32,65135956
	DM30-6	31,54084	34,54756165	NE	27,09753799	33,75973511
	DM30-7	28,971943	31,72974396	NE	25,86524963	31,75543594
	DM30-8	30,05162621	30,43880844	NE	26,25998116	32,65244675
	NDS30-1	31,427372	33,78091812	NE	30,07614517	34,54193878
	NDS30-2	36,381935	34,36175537	34,73279572	30,52637482	33,56607437
	NDS30-3	27,317463	30,16884804	NE	26,29711914	30,43796921

	NDS30-4	27,479073	30,6429863	35,57639313	26,63085938	30,62402725
	NDS30-5	32,52370453	29,51222229	NE	25,47675323	30,53217697
	NDS30-6	31,14347839	32,08449936	NE	26,61161041	34,8458786
	DM30-1	31,27412	34,52074051	NE	27,84400177	31,53963852
	DM30-2	32,991077	34,68645477	36,61274338	28,86483192	NE
	DM30-3	37,298954	NE	NE	34,31935883	NE
	DM30-4	35,08029	NE	NE	32,7063446	NE
	DM30-5	34,14043808	NE	NE	NE	34,02513885
	DM30-6	35,659737	NE	NE	33,36937332	NE
Rim	DM30-7	30,764107	NE	NE	30,53927994	33,06829834
	DM30-8	29,69210815	34,7585144	32,71249008	28,96990204	31,36323166
	NDS30-1	31,280132	33,51757431	35,89225388	36,38698196	NE
	NDS30-2	30,352713	NE	NE	35,34963226	NE
	NDS30-3	30,156494	33,97267151	NE	27,54672623	29,58517075
	NDS30-4	31,108461	33,82926559	NE	29,47044182	30,4120636
	NDS30-5	30,78092766	NE	34,34976959	26,83312416	32,07322311
	NDS30-6	29,49404907	NE	34,47018814	26,10942078	32,23060226
	DM30-1	33,48993683	NE	32,411983	33,422672	34,30681
	DM30-2	31,52414513	27,719131	31,829918	33,42641	33,331017
	DM30-3	31,95358276	36,253597	34,803223	30,551098	34,366333
	DM30-4	31,624529	35,61665344	33,289635	35,45089	NE
	DM30-5	37,34568405	35,901016	33,59369	34,94619	32,417316
	DM30-6	31,1158371	31,523472	33,018467	36,95848	28,543007
	DM30-7	32,52493286	30,49953651	32,34415436	NE	29,96485138
Sangue	DM30-8	29,4215374	31,56151199	29,46360779	29,83970642	32,24677277
	NDS30-1	27,57139778	NE	NE	32,669926	NE
	NDS30-2	27,6806488	36,20703888	34,844578	30,854656	NE
	NDS30-3	38,29730225	NE	NE	NE	33,758034
	NDS30-4	31,53600693	NE	NE	37,617447	35,136322
	NDS30-5	35,63544464	NE	NE	NE	NE
	NDS30-6	36,13992691	35,58192	NE	NE	NE
	DM30-1	35,028023	NE	NE	NE	NE

Urina	**DM30-2**	38,09850693	**NE**	**NE**	**NE**	**NE**
	**DM30-3**	35,558342	**NE**	**NE**	**NE**	**NE**
	**DM30-4**	34,751072	**NE**	**NE**	**NE**	**NE**
	**DM30-5**	32,743088	**NE**	**NE**	**NE**	**NE**
	**DM30-6**	35,20172	**NE**	**NE**	**NE**	**NE**
	**DM30-7**	33,264473	**NE**	**NE**	**NE**	**NE**
	**DM30-8**	30,20651436	36,76332092	33,62377167	36,83921051	34,67897797
	**NDS30-1**	33,49559	**NE**	**NE**	**NE**	**NE**
	**NDS30-2**	33,88241	**NE**	**NE**	**NE**	**NE**
	**NDS30-3**	33,817787	**NE**	**NE**	36,16558456	**NE**
	**NDS30-4**	32,284573	**NE**	**NE**	**NE**	**NE**
	**NDS30-5**	38,77697754	**NE**	**NE**	36,10826874	36,9862442
	**NDS30-6**	35,88370514	**NE**	**NE**	**NE**	**NE**
Coração	**DM40-1**	35,34549713	**NE**	**NE**	36,06498337	35,82475281
	**DM40-2**	33,62910461	32,48654938	37,05342102	30,04471016	33,33068085
	**DM40-3**	29,87382126	27,1897583	33,40861511	26,07508469	29,54026031
	**DM40-4**	29,87382126	34,25712967	34,60998535	33,26514435	35,24771118
	**DM40-5**	32,13407135	33,70717621	33,71962357	31,35220337	33,426651
	**NDS40-1**	31,07275772	31,81742668	34,87146378	29,75694275	35,70116806
	**NDS40-2**	32,02972412	**NE**	**NE**	34,72212219	36,97289658
	**NDS40-3**	32,34375	33,81221008	33,75255203	32,72080994	36,99131012
	**NDS40-4**	27,94813347	32,22982407	33,2882309	29,01842117	32,30204773
	**NDS40-5**	34,99194336	33,81119919	**NE**	37,03134537	35,42295074
	**NDS40-6**	32,04882813	**NE**	**NE**	36,28459167	
	**NDS40-7**	26,32171822	**NE**	33,29628372	25,81476593	
ebro	**DM40-1**	30,57872009	29,50057602	33,07300568	25,4002037	28,65978813
	**DM40-2**	30,06344604	33,03322601	**NE**	27,06168747	29,41062927
	**DM40-3**	32,88763428	**NE**	**NE**	**NE**	**NE**
	**DM40-4**	26,94410706	28,20530319	33,61248016	24,90303421	27,83940125
	**DM40-5**	26,33008575	27,81181526	**NE**	23,82565308	28,63843155
	**NDS40-1**	32,06661606	33,02695084	33,37633514	28,35159683	33,14640045

**Cére**	**NDS40-2**	27,62830353	28,978899	34,86861801	25,30800247	29,11141586
	**NDS40-3**	26,77223969	28,44791603	33,9025116	24,25284958	26,22633743
	**NDS40-4**	27,92449188	26,61519241	32,93590927	24,14455032	27,49023438
	**NDS40-5**	25,34077454	27,73345184	32,61088943	24,45212173	
	**NDS40-6**	31,96570015	32,92062759	**NE**	29,37766647	
	**NDS40-7**	27,73844528	29,77441406	34,52325439	26,00561905	
	**DM40-1**	32,54780579	34,30662918	**NE**	28,03886223	31,16959572
	**DM40-2**	33,8352356	**NE**	**NE**	29,31116104	
	**DM40-3**	30,15597916	**NE**	**NE**	27,42812538	
	**DM40-4**	29,61566162	**NE**	**NE**	26,04397392	
	**DM40-5**	27,5778389	**NE**	**NE**	26,91879654	
**Fígado**	**NDS40-1**	30,51399803	29,68942451	**NE**	**NE**	30,76734161
	**NDS40-2**	32,01500702	**NE**	**NE**	31,34770584	
	**NDS40-3**	32,50442505	**NE**	**NE**	31,21009064	
	**NDS40-4**	31,08504677	**NE**	**NE**	**NE**	
	**NDS40-5**	33,51292419	**NE**	**NE**	33,70035553	
	**NDS40-6**	33,35319138	29,60289001	**NE**	29,40767479	
	**NDS40-7**	29,88041878	29,03038025	**NE**	26,64088821	
	**DM40-1**	34,96910095	**NE**	**NE**	31,63096619	
	**DM40-2**	30,54432106	**NE**	**NE**	30,55776978	
	**DM40-3**	31,05576324	**NE**	**NE**	30,33856201	
	**DM40-4**	29,6181221	**NE**	**NE**	26,91659164	
	**DM40-5**	32,92323303	**NE**	**NE**	29,90320969	
**Rim**	**NDS40-1**	32,80050659	**NE**	**NE**	30,23693085	
	**NDS40-2**	29,93310165	**NE**	**NE**	28,35576057	
	**NDS40-3**	30,68966866	**NE**	**NE**	27,07654762	
	**NDS40-4**	33,7116394	**NE**	**NE**	34,01464081	
	**NDS40-5**	35,7841301	**NE**	**NE**	32,19058228	
	**NDS40-6**	**NE**				
	**NDS40-7**	30,60009575	34,95578766	**NE**	25,50953293	
	**DM40-1**	31,25572205	**NE**	**NE**	29,55294609	
	**DM40-2**	32,8332901	**NE**	**NE**	32,31338501	

Sangue	DM40-3	27,75123215	NE	NE	28,77784538	
	DM40-4	27,6050415	28,37990952	29,69122314	28,3586998	
	DM40-5	33,76778412	NE	33,72143555	34,16440582	
	NDS40-1	31,60757446	NE	NE	29,83714294	
	NDS40-2	31,65962601	NE	NE	32,8899765	
	NDS40-3	27,76667786	29,54859352	30,99131775	28,43104172	
	NDS40-4	28,18144226	29,57136726	31,57911873	29,07003212	
	NDS40-5	33,4961586	34,93780136	35,10650253	35,43222809	
	NDS40-6	31,70485687	33,06095123	34,02398682	32,36399078	
	NDS40-7	31,26807404	32,78431702	34,51887512	32,12477875	
Urina	DM40-1	35,4408493	NE	NE	NE	NE
	DM40-2	35,23627472	NE	NE	37,13093185	NE
	DM40-3	34,68699646	NE	NE	NE	NE
	DM40-4	35,44897842	NE	NE	28,3586998	NE
	DM40-5	34,50245667	NE	NE	36,51391602	NE
	NDS40-1	37,82275391	NE	NE	NE	NE
	NDS40-2	31,68549347	NE	33,97755814	NE	NE
	NDS40-3	35,35892487	NE	NE	NE	NE
	NDS40-4	33,36891937	NE	NE	38,45373154	NE
	NDS40-5	32,88208771	NE	NE	NE	NE
	NDS40-6	34,87374878	NE	NE	NE	NE
	NDS40-7	33,80833435	NE	NE	NE	NE
